# Biocontrol potential and action mechanism of *Bacillus amyloliquefaciens* DB2 on *Bipolaris sorokiniana*

**DOI:** 10.3389/fmicb.2023.1149363

**Published:** 2023-04-06

**Authors:** Pengyu Luan, Yanjie Yi, Yifan Huang, Liuqing Cui, Zhipeng Hou, Lijuan Zhu, Xiujuan Ren, Shao Jia, Yang Liu

**Affiliations:** ^1^School of Biological Engineering, Henan University of Technology, Zhengzhou, China; ^2^Key Laboratory of Functional Molecules for Biomedical Research, Zhengzhou, China

**Keywords:** *Bacillus amyloliquefaciens* DB2, *Bipolaris sorokiniana*, biocontrol potential, antifungal mechanism, plant growth-promotion

## Abstract

**Introduction:**

*Bipolaris sorokiniana* is the popular pathogenic fungi fungus which lead to common root rot and leaf spot on wheat. Generally, chemical fungicides are used to control diseases. However, the environmental pollution resulting from fungicides should not be ignored. It is important to study the mode of antagonistic action between biocontrol microbes and plant pathogens to design efficient biocontrol strategies.

**Results:**

An antagonistic bacterium DB2 was isolated and identified as *Bacillus amyloliquefaciens*. The inhibition rate of cell-free culture filtrate (*CF*, 20%, v/v) of DB2 against *B. sorokiniana* reached 92.67%. Light microscopy and scanning electron microscopy (SEM) showed that the *CF* significantly altered the mycelial morphology of *B. sorokiniana* and disrupted cellular integrity. Fluorescence microscopy showed that culture filtrate destroyed mycelial cell membrane integrity, decreased the mitochondrial transmembrane potential, induced reactive oxygen species (ROS) accumulation, and nuclear damage which caused cell death in *B. sorokiniana*. Moreover, the strain exhibited considerable production of protease and amylase, and showed a significant siderophore and indole-3-acetic acid (IAA) production. In the detached leaves and potted plants control assay, *B. amyloliquefacien* DB2 had remarkable inhibition activity against *B. sorokiniana* and the pot control efficacy was 75.22%. Furthermore, DB2 suspension had a significant promotion for wheat seedlings growth.

**Conclusion:**

*B. amyloliquefaciens* DB2 can be taken as a potential biocontrol agent to inhibit *B. sorokiniana* on wheat and promote wheat growth.

## 1. Introduction

Wheat (*Triticum aestivum* L.) is the most important and widely planted global crop. According to the Food and Agriculture Organization of the United Nations (FAOSTAT: http://www.fao.org/faostat/en/) data, China produces more cereal crops, especially wheat with an estimated production of around 134 million tons, than any country in the world. However, wheat diseases caused by pathogenic fungi are the major limiting factors seriously affecting global wheat (Lu et al., 2020). Root and stem rot diseases of wheat caused a great loss of wheat yield and are substantial problems for crop planting. *Bipolaris sorokiniana* is a soil-borne pathogen known to cause common root rot and foliar blight in different wheat varieties, and is among the most destructive pathogens for wheat (Kumar et al., 2002; Zhang et al., 2020). In case of serious infection, more than 80% yield loss can be caused by *B. sorokiniana* under high-temperature and high-humidity environment (Kumar et al., 2009; Kaur et al., 2021). With the warming of climate and crop rotation, *B. sorokiniana* infects wheat increasingly and may be defined as the major pathogen in the wheat planting area of North China (Cohen and Leach, 2020; Su et al., 2021).

For infection, *B. sorokiniana* asexual conidiospore germinates on the surface of wheat leaves and forms an appressorium, then a penetration mycelia grows (Villa-Rodríguez et al., 2019). Also, the pathogen can survive in the soil to kill the root and crown of wheat seedlings, and its pathogenicity is associated with the production of toxins such as prehelminthosporol and helminthosporol (Kumar et al., 2001). Therefore, there is an urgent need to find sustainable and effective strategies for controlling this wheat disease. Chemical control is still the main way to prevent and control common root rot on wheat (Shcherbakova et al., 2018). It is reported that most chemical fungicides have excellent protective and curative power against a wide spectrum of crop diseases especially carbendazim and triazole fungicides (Bai et al., 2020). Unfortunately, frequent usage of chemical fungicides cause soil pollution, toxicity to humans, and the emergence of resistant strains (Manhas and Kaur, 2016). Due to limitations associated with chemical fungicide application, breeding wheat varieties with high levels of resistance is the most desirable method of control (Sari et al., 2019). However, it has been challenging to develop complete resistance cultivars. Up to now, biocontrol strategies have attracted huge scientific attention because they are environmentally friendly (Hanif et al., 2019).

Several microorganisms have been reported for the biocontrol of wheat diseases and promote plant growth (Lugtenberg and Kamilova, 2009). A successful antagonist strain *Chaetomium* sp. was earlier found to inhibit *B. sorokiniana* and *Fusarium culmorum* (Knudsen et al., 1995). Recently, *Bacillus subtilis* TE3 and *B. amyloliquefaciens* XZ34-1 were verified to be effective against *B. sorokiniana* (Villa-Rodriguez et al., 2021; Yi et al., 2021). Furthermore, the bio-efficacy of *Bacillus safensis*, *Chaetomium globosum*, and *Ochrobactrum pseudogrignonense* under *in vitro* and *in vivo*, and mechanism of antagonism also have been studied (Aggarwall et al., 2004; Al-Sadi, 2021). Nevertheless, the numbers of biocontrol agents are still limited nowadays. Currently, *Bacillus* spp. is widely used and developed in agriculture, which has the advantage over other biocontrol microorganisms because of producing various antifungal compounds and strong tolerance to extreme conditions changes (Su et al., 2021). Among them, *B. amyloliquefaciens* has a prominent ability to defeat plant pathogens (Jin et al., 2017; Awan et al., 2022). A previous study indicated that different strains of *B. amyloliquefaciens* can produce various levels of indole-3-acetic acid (IAA), extracellular enzymes, antibiotic compounds to protect plants from soil-borne fungal pathogen infection and promote plant growth (Belbahri et al., 2017). For instance, *B. amyloliquefaciens* SN13 is demonstrated to act as a biocontrol agent and enhance the immune response against *Rhizoctonia solani* in rice (Srivastava et al., 2016). *Bacillus amyloliquefaciens* strain SQR9 can inhibit the growth of *Fusarium oxysporum* in the cucumber rhizosphere (Xu et al., 2014). *Bacillus amyloliquefaciens* can produce antifungal substances to infect the normal growth of pathogens and lead to cellular necrosis and apoptosis because of mitochondrial damage and disruption of cell membranes (Li et al., 2016). *Bacillus amyloliquefaciens* LYZ69 induced reactive oxygen species accumulation and caused apoptosis-like cell death in *C. truncatum* (Hu et al., 2021). Despite this, the mode of antagonistic action between *B. amyloliquefaciens* and *B. sorokiniana* still needs to be studied in the future.

In the present study, the main objectives of this study were exploring the antifungal strain, investigating the possible action mode of *B. amyloliquefaciens* DB2 against *B. sorokiniana*, and evaluating the effects of DB2 culture filtrate on the pathogen control and growth promotion for wheat. This work will provide an effective biological agent to control *B. sorokiniana* and promote wheat growth.

## 2. Materials and methods

### 2.1. Materials

Soil samples for isolating strains were collected from the rhizosphere soil of wheat in Zhengzhou City, Henan province. The pathogenic fungus *B. sorokiniana* was kindly supplied by the Institute of Plant Protection, Henan Academy of Agriculture Sciences. Wheat cultivar “Zhengmai 366,” highly sensitive to *B. sorokiniana*, was used in the potted plants experiment. Wheat seeds were provided by the Institute of Wheat Research, Henan Academy of Agricultural Sciences.

### 2.2. Isolation and identification of antagonistic bacteria

Antagonistic strains were isolated and screened according to the method previously described by Wang et al. (2018). Soil samples were serially diluted up to 10^-6^ with sterile distilled water and spread on Luria-Bertani (LB) agar medium and incubated at 37°C for 48 h. The colonies with different morphology characteristics were selected for purifying, and their antifungal activities to *B. sorokiniana* were tested on potato dextrose agar (PDA) medium plates by plate confrontation method (Li et al., 2019).

Antagonistic strain DB2 was screened out, and the colony morphology, physiological, and biochemical characteristics were analyzed according to *Bergey*’s manual of systematic bacteriology (Bergey, 1994).

16S rDNA sequence analysis was performed for molecular identification of stain DB2. The genomic DNA was isolated using the modified CTAB method (Kumari et al., 2015). The partial sequence of 16S rDNA was amplified by PCR using bacterial universal primer pairs of 16SF (5′-AGAGTTTGATCATGGCTCAG-3′) and 16SR (5′-ACGGTTACCTTGTTACGACTT-3′; Weisburg et al., 1991). PCR conditions were carried out according to the protocol of Yi et al. (2021). The PCR products were sent to Bo Maide Biotech Co., Ltd. (Beijing, China) for sequencing. The sequences were submitted to NCBI^1^ under the accession number MZ664342 and compared using BLAST tool. Phylogenetic tree was constructed using the Neighbor-joining method of MEGA 11.0 with bootstrap values based on 1,000 replications (Zheng et al., 2021).

### 2.3. *In vitro* antagonistic activity of culture filtrate

Antagonistic strain was inoculated into the basal fermentation medium (0.5 g glucose, 2 g peptone, 1.25 g MgSO_4_, 0.2 g K_2_HPO_4_, 5 g yeast extract, 100 mL distilled water, and pH7) and cultured for 48 h at 37°C with shaking at 180 rpm, followed by centrifuging at 10,000 rpm for 10 min and filtering through 0.22 μm filter to obtain the cell-free culture filtrate.

Effects of culture filtrate (*CF*) on conidial germination of *B. sorokiniana* were tested using the micro-bioassay method in 24 well plates (Villa-Rodriguez et al., 2021). *Bipolaris sorokiniana* was added into 5 mL of sterile saline solution (0.9%) containing Tween-20 surfactant (polyoxyethylene sorbitan monooleate) and spread with a Drigalski loop (Monteiro et al., 2017). The conidia of *B. sorokiniana* were grown in potato dextrose broth (PDB) and diluted with distilled water, and its final concentration was determined with a hemocytometer. Next, 100 μL conidia suspension (10^5^ conidia/mL) of *B. sorokiniana* were inoculated into each well containing: (1) 700 μL PDB + 200 μL *CF* (to reach a final concentration of 20% v/v); (2) 800 μL PDB + 100 μL *CF* (to reach a final concentration of 10% v/v); and (3) 900 μL PDB as the control group. The wells were sealed and cultured in the dark at 28°C for 72 h for conidia observing under the optical microscopy. The fungal mycelia were harvested from well plates by centrifugation at 8,000 × *rpm* for 15 min. The pellet was washed with sterile water, and the dry weight was determined after heating in the oven at 105°C until a constant weight. Dry weight of fungal biomass was recorded and the inhibition effect was calculated using the following formula. The experiment was conducted with three replicates.


(1) $$\text{Inhibition rate}\;\left(\%\right)=\frac{\left({\text{FB}}_{\text{CK}}-{\text{FB}}_{\text{CF}}\right)}{{\text{FB}}_{\text{CK}}}\times 100$$

Where FB_CK_ represents fungal dry weight in control and FB_CF_ represents fungal dry weight under *CF* treatment.

### 2.4. Effects of culture filtrate on mycelial morphology of *Bipolaris sorokiniana*

The mycelial pellets of *B. sorokiniana* were recovered and washed with 0.01 M phosphate buffered saline (PBS, pH 7.2) after treatment with *CF* (20% v/v) for 12 h. Mycelial pellets were stained with 0.4% trypan blue (C0040, Solarbio) for 3 min and observed by revolve fluorescence microscopy (Echo-Labs Revolve, United States). Moreover, the mycelia were prepared for observing by scanning electron microscopy (SEM) according to a previously reported method by Bahena-Garrido et al. (2019). First, the mycelia were fixed with 2.5% glutaraldehyde solution, dehydrated with 30–100% ethanol solution, and resuspended in tert-butyl alcohol. Next, the mycelial was sequentially treated with an ethanol and isoamyl acetate (V/V = 1/1) mixture and isoamyl acetate, followed by critical point drying. Mycelial morphology observation of *B. sorokiniana* was performed with a scanning electron microscope (Hitachi SU8010, Tokyo, Japan).

### 2.5. Determination of culture filtrate on cell membrane integrity in pathogenic mycelia

For investigating the potential of *CF* to induce cell death of *B. sorokiniana*, the mycelial cell membrane integrity was evaluated (Kim et al., 2017). Briefly, *B. sorokiniana* were incubated for an additional 24 h in PDB supplemented with the cell-free culture filtrate (1:1, v/v). The mycelial cell without treatment was set as control. Mycelia were harvested and stained with 10 μg/mL propidium iodide staining solution (PI, Biyuntian Biotech) at 25°C in the dark for 10 min. Fluorescence signals were detected using revolve fluorescence microscopy (Echo-Labs Revolve, United States). Each treatment was repeated three times.

Mycelia were inoculated into 150 mL PDB and incubated for 72 h. And the culture were supplemented with 20 and 10% *CF* (v/v) followed by cultivating for 0, 2, 4, 6, 8, 10, and 12 h, and cultures without *CF* served as control. The pathogenic fungi culture were collected after centrifuging (6,000 rpm) for 5 min. The conductivity of the pathogenic culture with different treatment time were measured to determine the cell membrane leakage of *B. sorokiniana* (Shao et al., 2015; Lin et al., 2018). The ergosterol content in mycelial plasma membrane of *B. sorokiniana* treated with *CF* (1:1 v/v) for 48 h was determined as previously described by Qin et al. (2022).

### 2.6. DNA damage assay

To assess the nuclear damage of *B. sorokiniana*, the mycelial cells were stained with 10 μL of 1 μg/mL 49, 6-diamidino-2-phenylindole (DAPI; 10 μg/mL in PBS; BiYunTian Biotechnology, China) for 15 min and observed with revolve fluorescence microscopy (Echo-Labs Revolve, United States). Meanwhile, extracellular DNA was also measured to reflect the cell content outflow of the *CF* treated mycelia for 0, 2, 4, 6, 8, 10, and 12 h. Briefly, an UV–visible spectroscopy spectrometer was used to measure the DNA content in the solution of each treatment group at 260 nm. Each treatment was repeated for three times.

### 2.7. Mitochondrial membrane potential assay

To detect the mitochondrial membrane potential of *B. sorokiniana*, mycelia were stained with 5,59,6,69-tetrachloro-1,19,3,39-tetraethylbenzimidazolocarbocyanine iodide (JC-1 dye; 20 μg/mL; Biyuntian Biotechnology, China) using the modified method described by Chen et al. (2021). After incubation for 12 h at 28°C with culture filtrate, mycelia were stained with JC-1 for 30 min in the dark and observed.

### 2.8. Intracellular reactive oxygen species accumulation assay

Accumulation of ROS in *B. sorokiniana* attributable to treatment with the culture filtrates for 12 h was determined by staining the mycelial cells with 20 μM 2′,7′-Dichlorodihydrofluorescein diacetate (DCFH-DA; Molecular Probes, Biyuntian, China) at 25°C in the dark for 30 min, then, washed with PBS three times and observed with revolve fluorescence microscopy (Echo-Labs Revolve, United States) after DCFH-DA removal.

### 2.9. Detection of hydrolase and PGP properties of *Bacillus amyloliquefaciens* DB2

Amylase, protease, and cellulase activities were determined by starch agar, skim milk agar, and carboxymethylcellulose agar, respectively (Waithaka et al., 2017; Khalil et al., 2021; Xu et al., 2022). Plant growth-promotion (PGP) tests include indole acetic acid (IAA) production, siderophores production, and phosphate solubilization (Shcherbakova et al., 2018; Lotfi et al., 2022; Mirskaya et al., 2022). The ability of bacterium to produce IAA was determined by Salkowski’s colorimetric method. After incubation, the broth was centrifuged and mixed with Salkowski reagent to observe the development of pink color. The bacterium was incubated at 37°C for 7 days and change of the blue color in the medium surrounding the colony was scored as positive for production of siderophores. For determining the inorganic or organic phosphate solubilization capacity, the bacterium was inoculated on the Pikovskaya agar containing tricalcium phosphate. After 7 days of incubation at 37°C, the formation of clearing zones was evaluated and indicated the utilization of tricalcium phosphate present in the agar medium.

### 2.10. Biocontrol effects of culture filtrate in greenhouse experiments

The control effect of *CF* on detached leaves of wheat seedlings (Zhengmai 366) was evaluated according to the method by Mishra et al. (2014). Seeds of wheat were soaked in 1% sodium hypochlorite for 15 min, rinsed with sterile water for five times. Seed germination was placed in Petri dishes containing moist filter paper and performed in the dark at 26°C for 24 h. The germinated seeds were sowed in a sterile plastic basin (110 mm × 80 mm), and cultured at 25 ± 2°C, with the relative humidity of 50 ± 5% and the light: dark (L:D) ratio of 14:10 h under the greenhouse. When the wheat leaves grew to one leaf and one terminal bud stage, the leaves (6 cm length) were cut off and soaked in 10 mL liquid as (1) sterile water, (2) *CF* of DB2, and (3) 50% triadimefon at Petri dishes for 1 h, respectively. Subsequently, 6-mm-diameter plugs from fresh cultures of *B. sorokiniana* were inoculated onto leaves. There were six leaves on each plate and three plates in each treatment. Disease severity was inferred from the lesion size on the leaves after 5 days of infection.

The potted plants control assay was performed under the greenhouse conditions using wheat Zhengmai 366. 25 seeds were sown in each pot. The germination conditions were same as described above in detached leaves control tests. When the wheat grew to one leaf and one terminal bud stage, pathogenic conidial suspension (10^6^ conidia/mL) was prepared in sterile water and inoculated on potted wheat. After 7 days, wheat roots in each pot were irrigated with 20 mL *CF*. 50% triadimefonl (positive control), and sterile water (negative control). Every treatment include four pots and the biocontrol experiment conducted thrice.

Disease severity was evaluated when the leaves in the control group were fully infected, and followed the scoring standard by Yi et al. (2021). The disease incidence rate (DIR), disease index (DI), and control efficacy (CE) were using the following formulas:


(2) $$\text{DIR}\;\left(\%\right)=\frac{n}{N}\times 100$$

Where *n* is the number of infected plants, *N* is the total number of investigated plants.


(3) $$\text{DI}=\sum \left[\frac{\left(N_{i}\times i\right)}{\left(N\times 4\right)}\right]\times 100$$

Where *N*_i_ denotes the number of infected plants of a certain severity, *i* denotes a certain severity, and *N* is the total number of investigated plants.


(4) $$\text{CE}\;\left(\%\right)=\left[\frac{\left({\text{DI}}_{\text{CK}}-{\text{DI}}_{T}\right)}{{\text{DI}}_{\text{CK}}}\right]\times 100$$

Where DI_CK_ indicates the disease index of the control group, and DI_T_ indicates the disease index of the treatment group.

### 2.11. Growth-promotion for wheat under greenhouse

The plant growth-promoting efficacy of DB2 strain on wheat was hydroponically cultured in a greenhouse. In a short, the wheat seeds were surface sterilized with 1% sodium hypochlorite for 15 min and washed five times with sterilized distilled water. Suspension of DB2 was prepared with sterile water and adjusted to different concentrations (10^6^, 10^7^, and 10^8^ CFU/mL). 25 wheat seeds were placed in 12 mm culture dishes in incubator at 26 ± 2°C, with the light:dark (L:D) ratio of 14:10 h. The seedlings were randomly divided into six groups and irrigated with 10 mL of LB medium, sterile water, culture filtrate, and DB2 suspension (10^6^, 10^7^, and 10^8^ CFU/mL) at roots, respectively. The experiments were conducted in a randomized complete block design with four culture dishes per treatment. The growth parameters of wheat seedlings were measured after 7 days, including plant height (mm), root length (mm), plant fresh weight, and dry weight (Yue et al., 2019). The experiment was repeated for three times.

### 2.12. Statistical analysis

Data were collected by Excel 2016 (Microsoft Corporation, United States) for calculating standard errors (SE) and standard deviations (SD). Meanwhile, ANOVA significance was analyzed through multiple comparisons with Duncan’s multiple range test using SPSS 22.0 (SPSS Inc., Chicago, IL). *p* < 0.05 indicates a significant difference.

## 3. Results

### 3.1. Identification of antagonistic strain DB2

A total of 80 culturable bacterial isolates were obtained from soil samples and examined for antagonistic activity to the *B. sorokiniana in vitro*. Among them, we found the strain DB2 demonstrated higher inhibitory activities against fungal growth. The colony morphologies showed strain DB2 is rod-shape and its surfaces are rough, opaque, and milky white. The physiological and biochemical characteristics of DB2 were summarized in Table 1. Nitrate reduction, citrate utilization, gram staining, glucose decomposition, and V-P tests are all positive. The catalase test and methyl red are negative.

**Table 1:** Morphological and biochemical characteristics of DB2.

Morphological and biochemical characteristics		DB2
Colony morphology	Gram’s reaction	Gram positive
	Shape	Rounded, rod-shaped
	Pigment	Creamy white
	Surface	Rough
	Margin	Irregular
	Opacity	Opaque
	Endospores	+
Biochemical tests	Nitrate reduction	+
	Citrate utilization	+
	V-P test	+
	Methyl red	−
	Glucose decomposition	+
	Catalase test	−

“+” positive; “−” negative.

**Figure 1:**
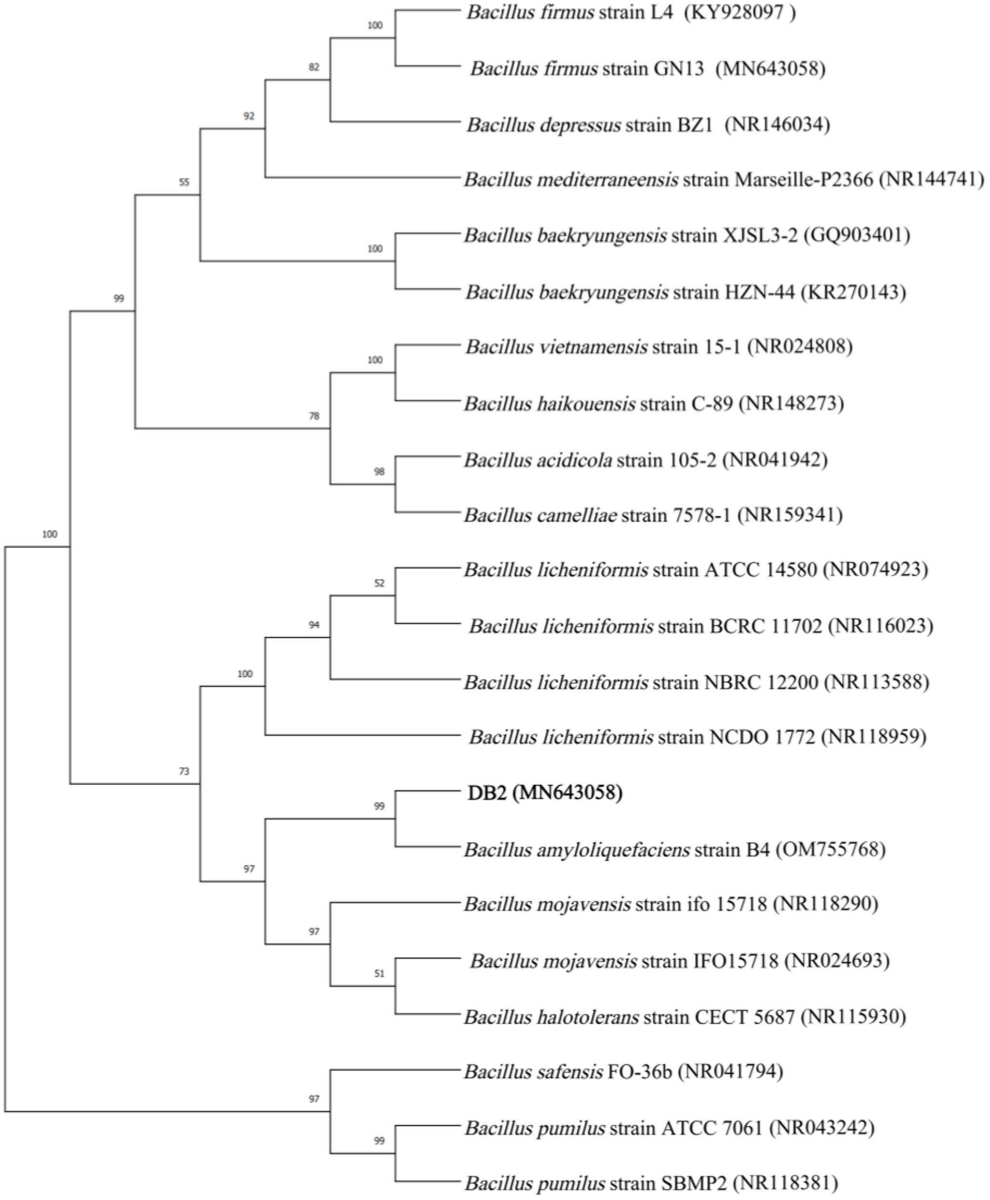
Phylogenetic tree of *Bacillus amyloliquefaciens* DB2 constructed on 16S rDNA sequence. The analysis was performed using the soft MEGA 11.0.

The 16S rDNA sequence of strain DB2 were submitted to GenBank and got the accession number of MZ664342. A phylogenetic tree was constructed based on 16S r DNA sequences from different strains (Figure 1). The results showed that DB2 and *B. amyloliquefaciens* strain B4 (OM755768) were in the same minimum branch. Moreover, the homology of 16S rDNA sequence of DB2 and *B. amyloliquefaciens* strain MPA (NR117946) was 99% according to the phylogenetic tree. Based on physiological, biochemical and molecular identification results, the strain DB2 was identified as *B. amyloliquefaciens* DB2.

### 3.2. *In vitro* inhibition of culture filtrate on *Bipolaris sorokiniana*

**Figure 2:**
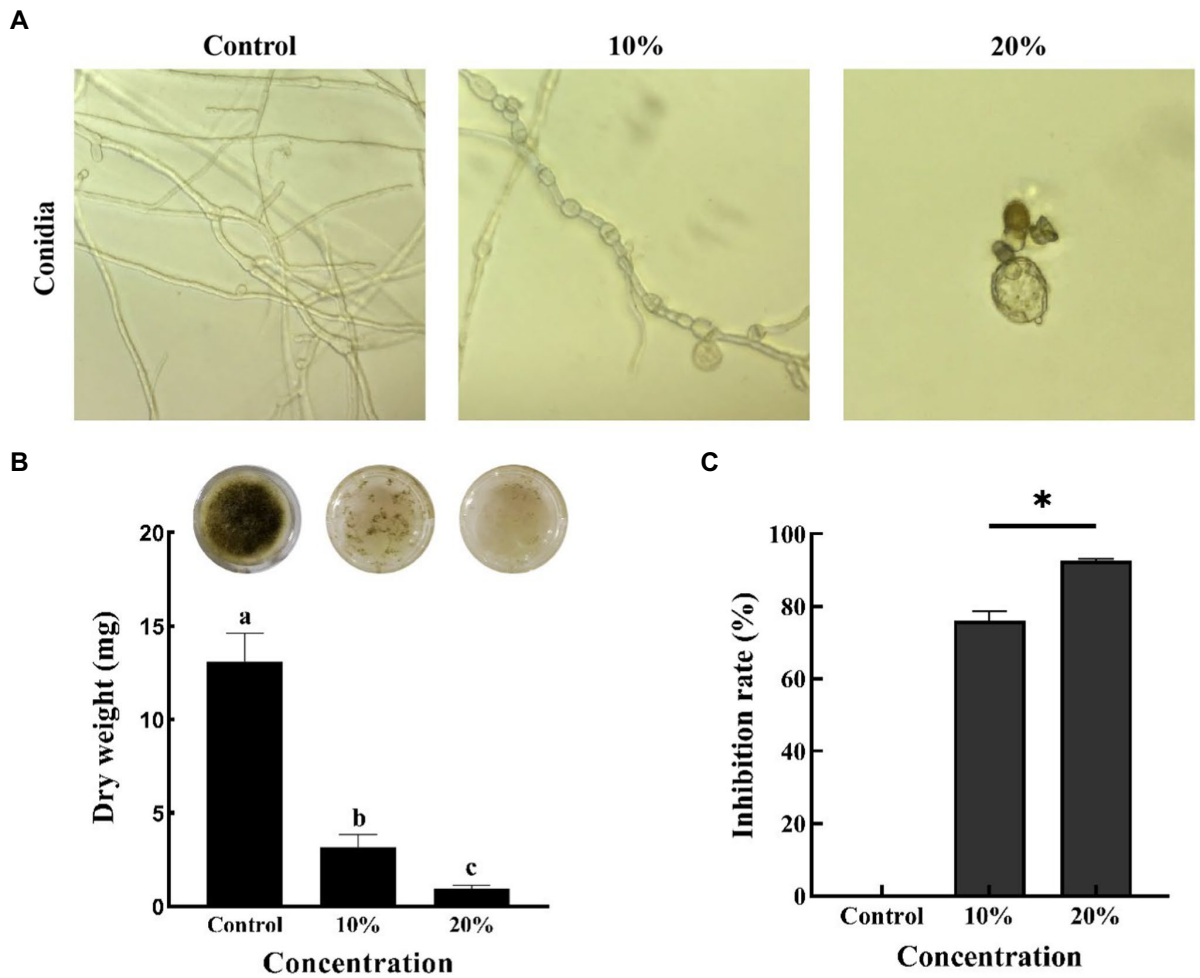
Inhibition of cell-free culture filtrate with different concentrations on *Bipolaris sorokiniana*. **(A)** Effect of cell-free culture filtrate on conidial germination of *Bipolaris sorokiniana*. **(B)** Effect of cell-free culture filtrate on dry weight of mycelium. **(C)** Inhibition rate of cell-free culture filtrate against *Bipolaris sorokiniana*. Error bars represent the standard deviation (*n* = 3). Different letters and ^*^ indicated a significant difference at the level of *p* < 0.05.

Microscopic observation showed the spores of *B. sorokiniana* appeared to be intact and grew normally in control group, whereas *CF* treatment resulted in abnormal germination of *B. sorokiniana* spores with round and vacuolar shape (Figure 2A). Furthermore, the *CF* reduced the mycelial dry weight of *B. sorokiniana* from 13.1 ± 1.5 to 0.96 ± 0.16 mg (Figure 2B), and mycelial growth inhibition rate was 92.67% (Figure 2C). Thus, *CF* can significantly inhibit the growth of mycelia of *B. sorokiniana*.

### 3.3. Mycelial morphological changes of *Bipolaris sorokiniana*

**Figure 3:**
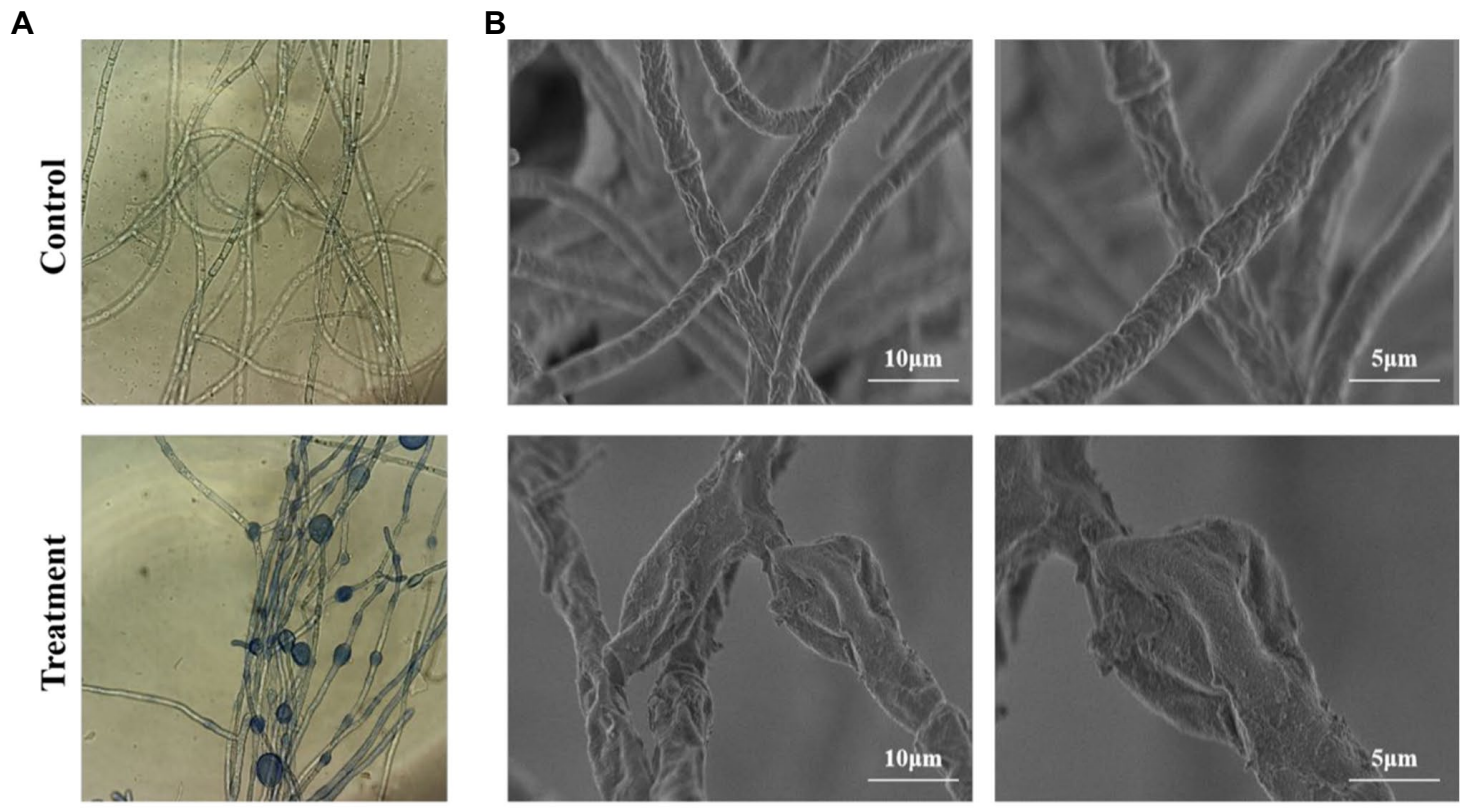
Effect of cell-free culture filtrate on the morphology of *Bipolaris sorokiniana*. **(A)** Mycelium morphology under optical microscope (×20), blue color represents dead fungi cells. **(B)** Mycelium ultrastructure under scanning electron microscope, scale bars = 10 and 5 μm. Control: untreated control group; Treatment: treated with cell-free culture filtrate produced by *Bacillus amyloliquefaciens* DB2.

The mycelia of *B. sorokiniana* treated with *CF* exhibited swollen and bulbous-like. Cell death was further investigated using trypan blue, which accumulated in dead cells. The swollen mycelia were filled with the blue dye (Figure 3A). However, the mycelia in the control were normal, well organized and not showed color changed. SEM observation showed the mycelial damage with *CF* treatment. The mycelia in the control group were normal and the surface was smooth, plump, and integrity. In the treatment group, the mycelia were loose, shrunken, and collapsed (Figure 3B). Overall, SEM and light microscope observation of the mycelial morphology was changed severely.

### 3.4. Effects of *CF* on membrane integrity of *Bipolaris sorokiniana*

**Figure 4:**
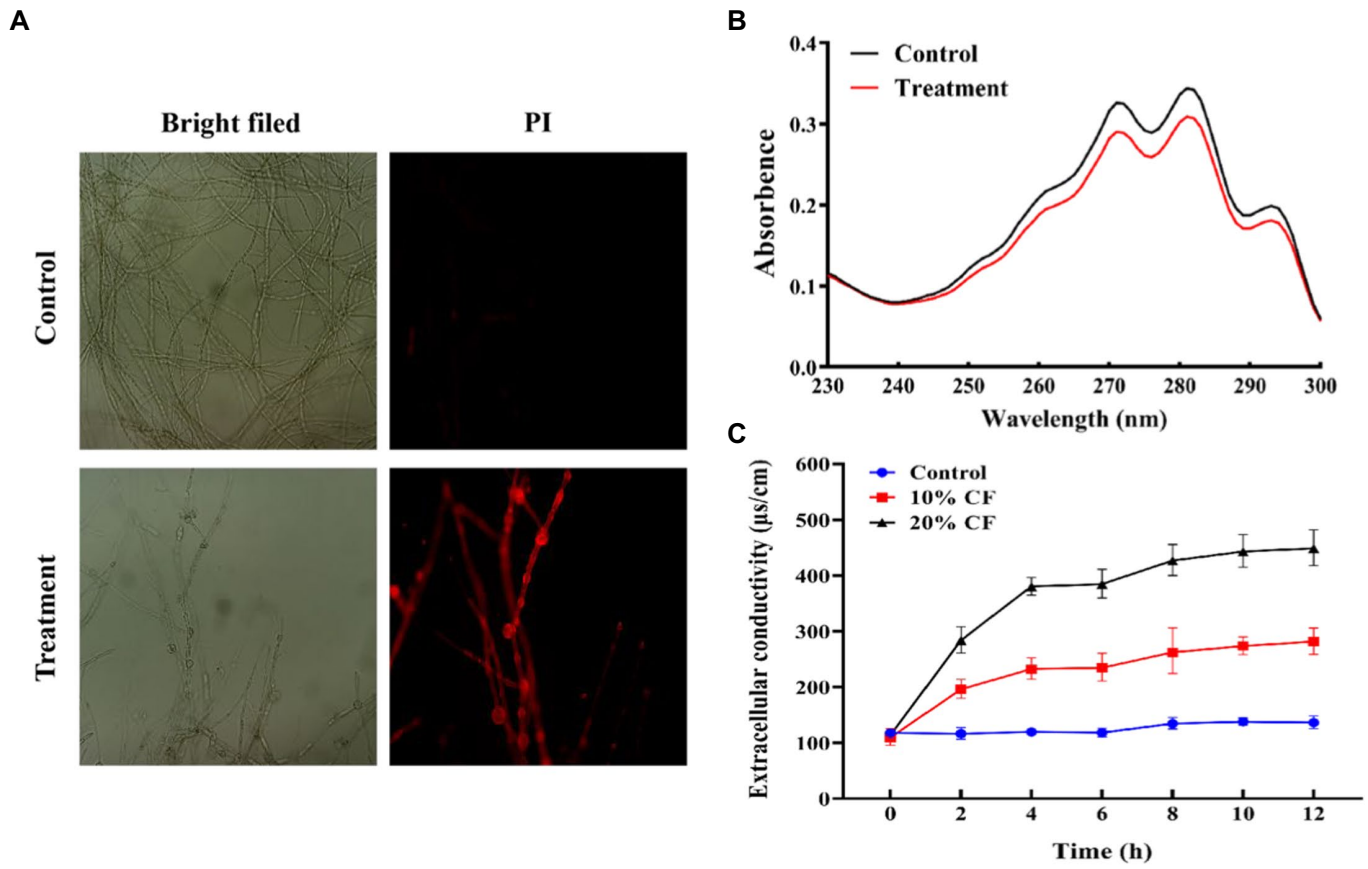
Effect of cell-free culture filtrate on membrane integrity of *Bipolaris sorokiniana* mycelium. **(A)** Fluorescence of mycelia stained with PI measured by revolve fluorescence microscopy (×20). **(B)** Ultraviolet scanning spectra of ergosterol content in the mycelium plasma membrane treated with cell-free culture filtrate. **(C)** Effects of cell-free culture filtrate on the electric conductivity of mycelium. Values are presented as mean ± SE (*n* = 3).

To investigate the possible mode of antifungal action of *CF* against *B. sorokiniana*, plasma membrane and the conductivity of *B. sorokiniana* were determined to assess membrane integrity and permeability. The PI-stained mycelia of *B. sorokiniana* exhibited more extensive red fluorescence in the *CF* treatment group than that in the control group (Figure 4A), indicating that *CF* treatment can lead to cell membrane injury. The ergosterol content was measured by UV-spectrophotometer through a unique spectral peak between 240 and 300 nm. The *CF* reduced ergosterol production significantly in both spectral peaks when compared to control (Figure 4B). *CF* have effectively inhibited fungal growth (biomass) by reducing ergosterol content. The extracellular conductivity in *B. sorokiniana* suspension was increased with exposure time and the concentrations of *CF* (Figure 4C). Moreover, the extracellular conductivity increased with the incubation time during the first 8 h. Afterward, the growth tended to slow down. These results showed that the membrane integrity of *B. sorokiniana* was damaged by *CF*.

### 3.5. Effects of *CF* on DNA fragmentation

**Figure 5:**
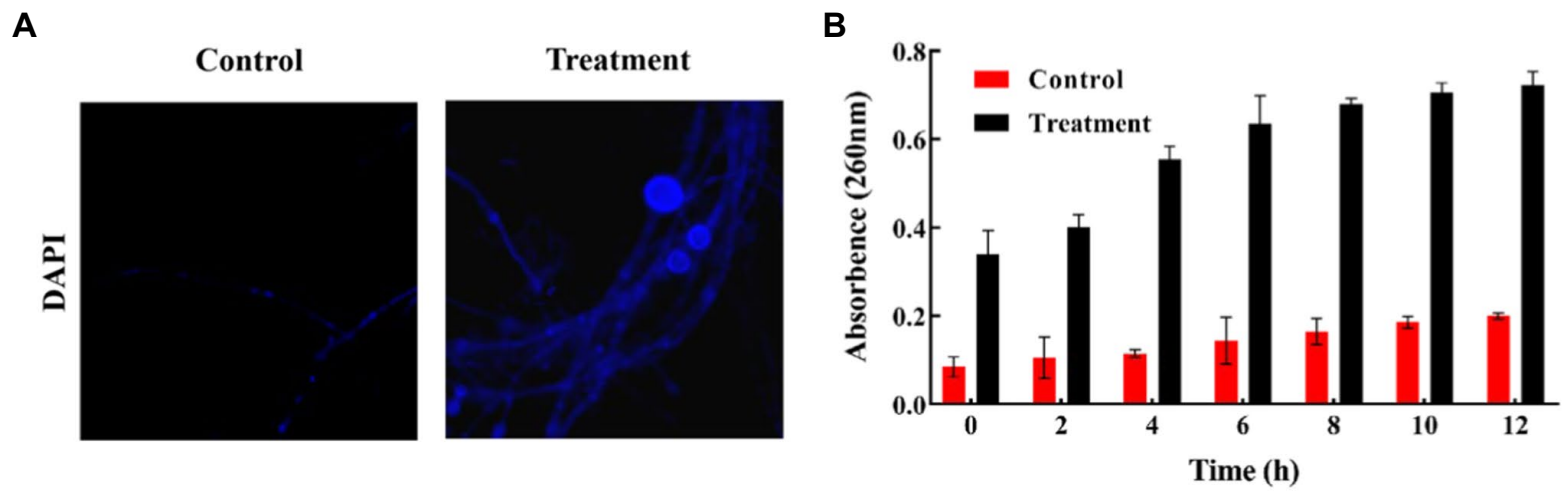
Effect of cell-free culture filtrate on the DNA fragmentation and leackages in *Bipolaris sorokiniana* cells. **(A)** cell-free culture filtrate produced by *Bacillus amyloliquefaciens* DB2 induced chromatin condensation in hyphal cells (×20). **(B)** Effects of cell-free culture filtrate on the nucleic acids of mycelium. Values are presented as mean ± SE (*n* = 3).

To further explore the inhibition mechanism of *CF*, the DAPI staining assays were performed to observe DNA and nuclear morphology. The mycelia treated with *CF* exhibited presenting diffuse nuclear staining compared with untreated mycelia (Figure 5A). These findings suggested that *CF* treatment damaged DNA in *B. sorokiniana* mycelia. After incubation of culture filtrate with *B. sorokiana* for 12 h, the OD_260_ of the centrifuged mycelial suspension was significantly higher than that of the control group (Figure 5B). The longer the incubation time with the culture filtrate was, the higher the OD_260_ value of the centrifuged mycelial suspension, indicating that the cell membrane integrity of *B. sorokiniana* was greatly damaged, which could consequently lead to high leakage of nucleic acids.

### 3.6. Effects of *CF* on mitochondrial membrane potential of *Bipolaris sorokiniana*

**Figure 6:**
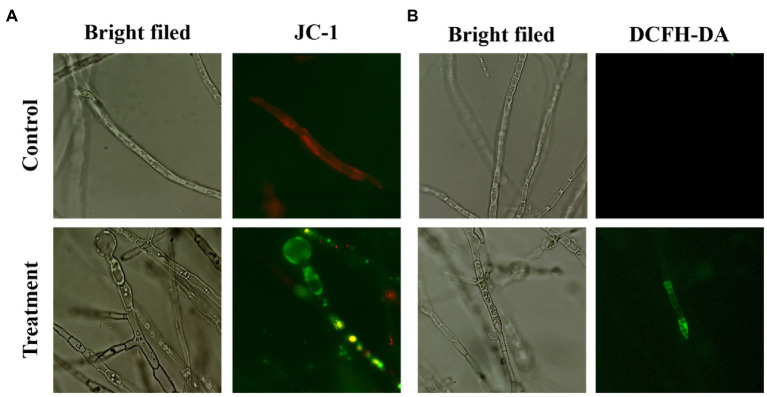
Effect of cell-free culture filtrate induced alteration of mitochondrial membrane potential (MMP) and reactive oxygen species (ROS) generation in *Bacillus amyloliquefaciens* cells. **(A)** Effect of cell-free culture filtrate on mitochondrial membrane potential of hyphal cells (×40). **(B)** Accumulation of reactive oxygen species in *Bipolaris sorokiniana* treated with cell-free culture filtrate. Cells were stained with DCFH-DA and observed with a revolve fluorescence microscopy (×40). PDB medium was used as a control, cell-free culture filtrate treated was used as a treatment.

To detect mitochondrial damage of the mycelia, the signal of the JC-1 staining was detected using fluorescence microscopy. The red fluorescence decreased and green fluorescence increased when mitochondrial damage occurs. The results showed that red fluorescence was aggregated in the untreated mycelia, whereas the cells treated with the *CF* exhibited a green color, suggesting that the *CF* caused reductions in the mitochondrial membrane potential of *B. sorokiniana* (Figure 6A).

### 3.7. Effects of *CF* on accumulation of ROS in *Bipolaris sorokiniana*

The burst generation of ROS could not only damage the cellular compound such as DNA and enzymes, but also increase the cell membrane permeability and lead to membrane structural damage. DCFH-DA was used to evaluate the level of oxidative stress of mycelia after different treatments. Fluorescence intensity represents the accumulation of ROS. The green fluorescence was observed in the *CF*-treated cells but not in the untreated cells, indicating that the accumulation of ROS was induced by the *CF* of *B. amyloliquefaciens* DB2 (Figure 6B).

### 3.8. Analysis of hydrolase and PGP traits of *Bacillus amyloliquefaciens* DB2

**Figure 7:**
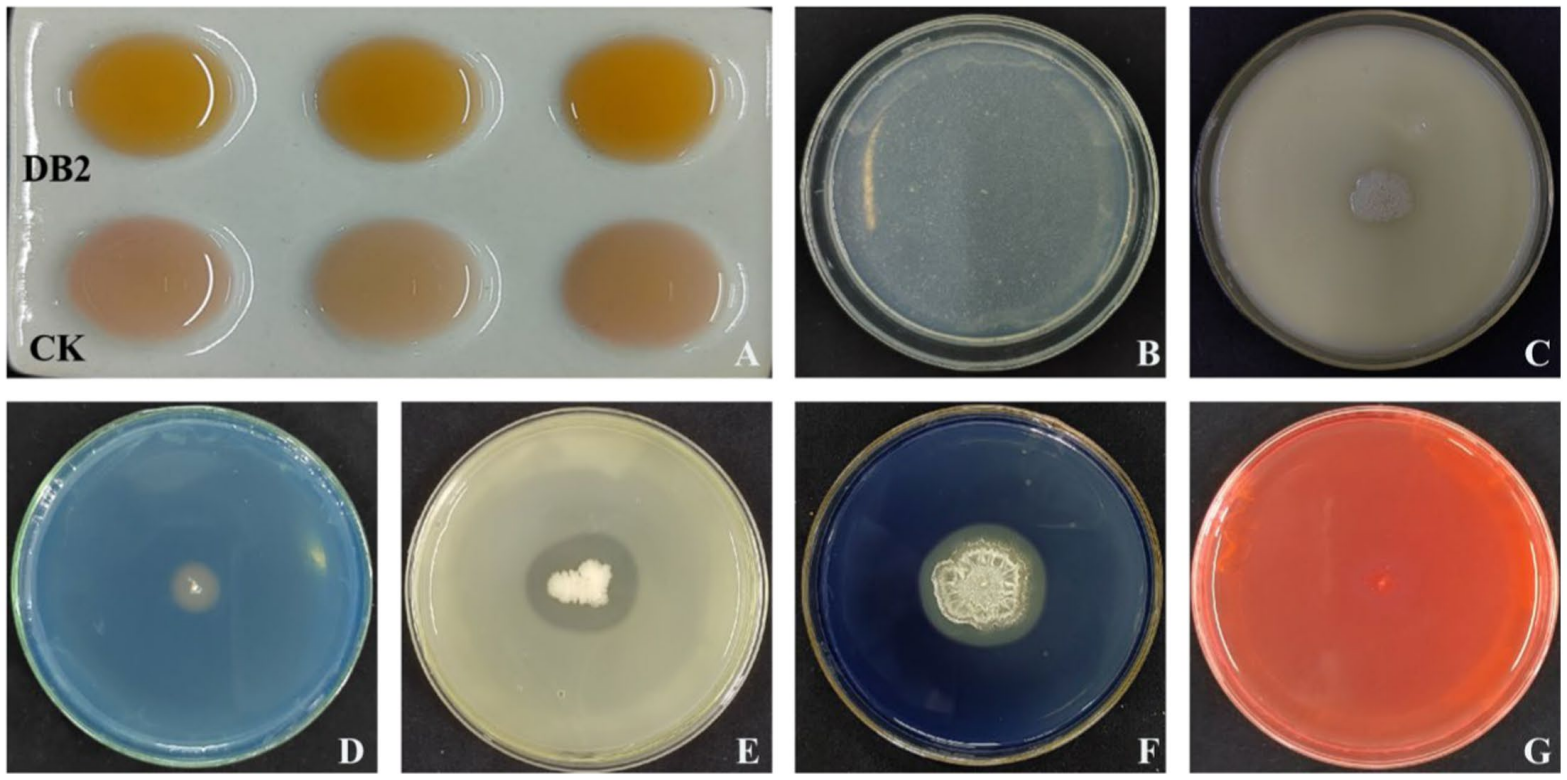
PGP and antifungal traits of strain DB2. **(A)** The upper indicating IAA production by color change. **(B)** The strain DB2 was negative on the inorganic phosphate solubilization test by no halo zone around colonies. **(C)** The strain DB2 was negative on the organic phosphate solubilization test by no halo zone around colonies. **(D)** Yellow-orange halos indicating siderophore production. **(E)** Protease activity indicated by a clear zone around strain DB2 colonies. **(F)** Amylase activity indicated by an obvious hydrolytic zone around strain DB2 colonies. **(G)** The strain was negative for cellulase activity by no zone of hydrolysis around strain DB2.

Antifungal substance determination showed that strain DB2 could produce amylase and protease due to the presence of a clear zone in a special medium. The absence of transparent halos on the test plates for cellulose indicated this strain was incapable to produce such enzymes (Figure 7). Plant growth-promoting (PGP) traits detection indicated *B. amyloliquefaciens* DB2 was able to produce siderophore and IAA, while not to solubilize phosphate (Figure 7).

### 3.9. Biocontrol effects of strain DB2 on common root rot of wheat

**Figure 8:**
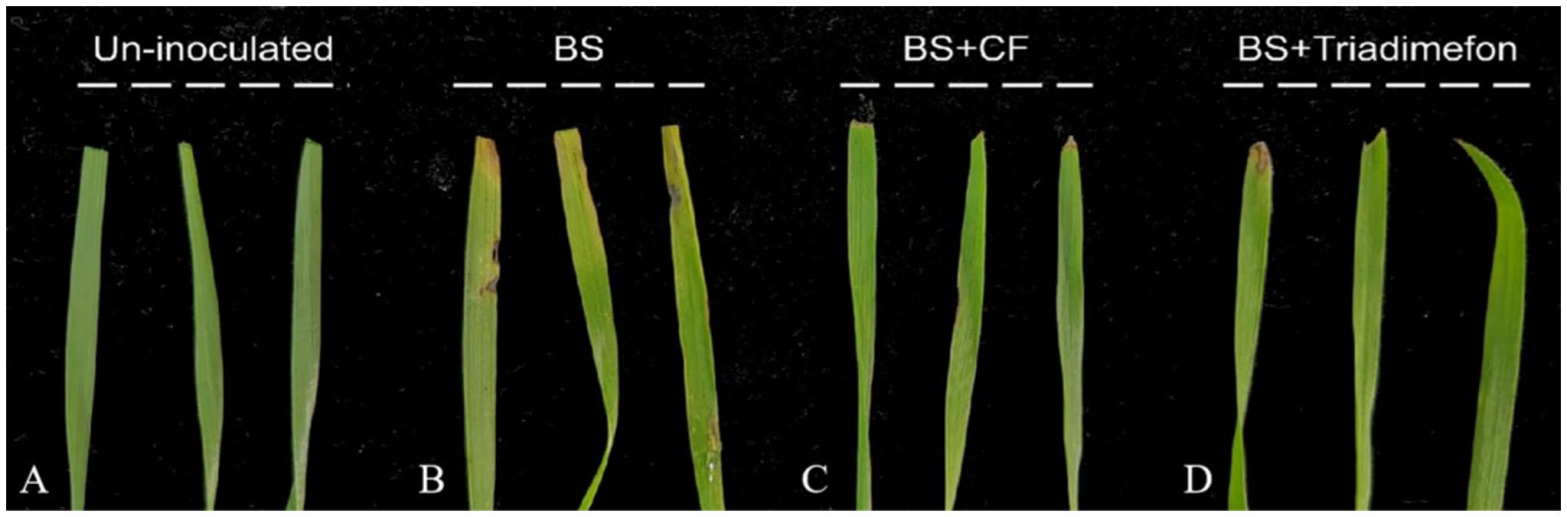
Control effect of DB2 on spot blotch in a detached leaf assay. **(A)** Sterile water treatment (Un-inoculated). **(B)** Only *Bipolaris sorokiniana* (BS) inoculation without cell-free culture filtrate (*CF*) treatment. **(C)** BS + cell-free culture filtrate treatment. **(D)** BS + Triadimefon treatment.

The biocontrol efficacy of strain DB2 against *B. sorokiniana* was evaluated using detached leaf and potted wheat control assay. At fifth day after infection, we evaluated the presence and area of necrotic lesions on wheat leaves. In the group inoculated with conidial suspension of *B. sorokiniana* alone, leaves showed the characteristic disease symptom of spot blotch. However, the *CF* and the triadimefon could significantly reduce the lesion area which appeared to be as healthy as control leaves (Figure 8). In the potted plants experiment, the visible disease incidence was observed in positive control. In *CF* and triadimefon treatment groups, disease incidence was significantly reduced with 35.20 and 39.20%, and the biocontrol efficacy reached 75.22 and 82.56%, respectively (Table 2). In summary, *CF* of *amyloliquefaciens* DB2 showed significantly control on common root rot of wheat.

**Table 2:** Control effect of DB2 on common root rot in the greenhouse.

Treatments	Disease incidence rate (%)	Disease index	Control efficacy (%)
Control	91.20 ± 3.35^a^	60.10 ± 1.79^a^	--
Triadimefon	35.20 ± 5.22^c^	10.45 ± 1.18^c^	82.56 ± 2.23^a^
*CF*	39.20 ± 2.19^b^	14.96 ± 4.45^b^	75.22 ± 6.72^b^

Statistical significance was determined using an independent sample *t*-test. ^z^Different uppercase letters in the same column indicate significant differences among treatments (*p* < 0.05). Standard errors of the mean values are presented after the ± symbol.

### 3.10. Plant growth promotion action of strain DB2 for wheat seedlings

The performance of the wheat root was far better than the control when bio-primed with the *B. amyloliquefaciens* DB2 suspensions. For wheat seeds, soaking with DB2 suspensions at different concentrations of 10^6^, 10^7^, and 10^8^ CFU/mL significantly promoted the growth of wheat seedlings (Table 3). Compared to the sterile water treatment group (control), 10^8^ CFU/mL DB2 suspension treatment increased the height, root length, fresh weight, and dry weight of wheat seedlings by 34.12, 90.27, 48.11, and 39.81%, respectively. In addition, LB liquid medium treatment caused a significant reduction in wheat growth including plant height and root length as compared to water treatment group. Interestingly, *CF* of *B. amyloliquefaciens* DB2 alleviated the impact of LB medium stress symptoms on the growth of wheat seedlings (Table 3).

**Table 3:** Effect of *Bacillus amyloliquefaciens* DB2 suspension on the growth of wheat seedlings.

Treatment	Height (mm^z^)	Root length (mm^z^)	Fresh weight (mg^z^)	Dry weight (mg^z^)
Sterile water	90.94 ± 0.63^e^	53.98 ± 0.47^d^	165.17 ± 1.22^e^	26.41 ± 1.76^c^
LB	53.94 ± 1.02^f^	32.40 ± 0.32^e^	104.43 ± 2.29^f^	23.00 ± 0.85^d^
*CF*	99.39 ± 0.41^d^	55.82 ± 0.49^c^	180.27 ± 1.00^d^	31.30 ± 0.66^b^
1 × 10^6^ CFU/mL	108.11 ± 1.76^c^	74.32 ± 0.49^b^	191.80 ± 0.89^c^	31.64 ± 0.58^b^
1 × 10^7^ CFU/mL	115.65 ± 1.05^b^	75.73 ± 1.15^b^	204.77 ± 0.70^b^	32.04 ± 2.22^b^
1 × 10^8^ CFU/mL	121.97 ± 1.12^a^	102.71 ± 1.22^a^	244.63 ± 2.03^a^	36.92 ± 1.05^a^

Statistical significance was determined using a Duncan’s multiple range test. ^z^Different uppercase letters in the same column indicate significant differences among treatments (*p* < 0.05). Standard errors of the mean values are presented after the ± symbol.

## 4. Discussion

*Bipolaris sorokiniana* is an important soil-borne plant pathogen causing root rot disease in various crops leading to agricultural economic losses. Biological control is considered as an environmentally-friendly, sustainable, and effective approach to control plant diseases. In the present study, the strain DB2 was isolated from the rhizosphere soil of wheat and identified as *B. amyloliquefaciens* based on its morphology and molecular characters. To further study the mechanism of *B. amyloliquefaciens* DB2 in controlling fungi, *B. sorokiniana* was used as an indicator. Here, we evaluate the antifungal activity of cell-free culture filtrate (*CF*) of *B. amyloliquefaciens* DB2 against *B. sorokiniana*. The results showed that *CF* could significantly inhibit the growth of mycelia and conidia germination, which reached an inhibition rate of 92.67%. This indicated that the antifungal activity of *CF* showed higher antagonists, which was similar to the results of Shemshura et al. (2020) Several lines of evidence suggest that *Bacillus* species can affect abnormalities in the mycelia of pathogens, causing cell wall lysis, breakage, granulation, and vacuolization (Chaurasia et al., 2005). Li et al. (2015) reported that bacteria-free filtrate from *Bacillus megaterium* strain L2 caused some of the germinal tubes to undergo vacuolization and even rupturing. A similar phenomenon was observed in *B. sorokiniana* after culture filtrate treatment. In this study, we found that the *CF* induced swollen, bulbous-like from the light microscope, and loose, shrunken, and collapsed in the surface of the mycelia under SEM.

In the investigation of the antifungal mechanism of *B. amyloliquefaciens* DB2 on *B. sorokiniana* at the cellular level, the *CF* could damage the mycelial cell membrane of *B. sorokiniana*. In this study, the data indicated that the *CF* can disrupt cell membranes resulting in PI staining accumulating in necrotic mycelial cells. Due to the disruption of *CF*, ergosterol content decreased and electrical conductivity increased by comparison with the control group. Thus, our results urther demonstrated that *CF* alters membrane permeability and integrity. Xu et al. (2019) has reported that the cell-free supernatant from *B.amyloliquefaciens* LZN01 caused the disruption of cell walls and membranes, the leakage of intracellular contents and the aggregation of organelles in *Fusarium oxysporum*. The changes in mitochondrial membrane potential (MMP) could play a role in the induction of cell metabolism. At the present study, the MMP of *B. sorokiniana* declined in mitochondrial function leading to enhanced ROS production. Next, the accumulation of excess ROS leads to damage to intracellular DNA and the efflux from cell membranes. Wang et al. (2020) has reported Iturin A extracted from *Bacillus subtilis* WL-2 affected *phytophthora infestans* triggering oxidative stress reactions and causing mitochondrial damage, including MMP. According to the previous study, the *CF* not only induced membrane damage, a disrupting membrane potential but also by interfering with energy metabolism in *B. sorokiniana*. These results indicated the biocontrol mechanism of strain DB2 was inducing cell death by disrupting the mycelial cell membrane of *B. sorokiniana* and affecting intracellular metabolic processes.

Previous studies reported *B. amyloliquefaciens* played a vital role in the crop protection and plant growth promotion (Yuan et al., 2012; El-Gremi et al., 2017). The plant growth promoting ability was also used as a second alternative or complementary means for plant protection. Our strain DB2 produced indole-3-acetic acid (IAA), siderophores, and major enzymes such as protease, amylase. Indole-3-acetic acid (IAA) can stimulate cell proliferation and elongation of plant roots to regulate the growth of plant roots (Gupta and Pandey, 2019). In addition, strain DB2 showed the ability to produce siderophores. It is common that siderophore producing bacteria could supplement iron to the plant even prevent the growth of the soil-borne pathogens (Hilgers et al., 2021). Generally, the bio-control bacteria can interact with pathogens through a series of mechanisms, including the production of antibiotics and lytic enzymes (Ben Slama et al., 2019). In a previous study, *B. amyloliquefaciens* SQR9 was reported as a growth-promoting activity in greenhouse experiments including indole-3-acetic acid (IAA) production and extracellular phytase (Shao et al., 2015). And *B. amyloliquefaciens* QSB-6 have a good inhibitory effect on *Fusarium* in the soil and can significantly promote plant root growth (Duan et al., 2021).

To further evaluate the ability of DB2 to control diseases, a detached wheat leaf assay was conducted. We found our strain had strong inhibitory activity against *B. sorokiniana* on wheat leaf. Moreover, the bio-control efficiency of the *CF* was further assessed in a pot experiment. It proved that preventive treatment result was very efficient in coping with the development of the infection by the *B. sorokiniana*. The other previous reports had studied the *Bacillus* strains can use to overcome common root rot diseases (Campanella et al., 2020; Villa-Rodriguez et al., 2021). Our strain DB2 has good application potential in the sustainable development of the agriculture.

During wheat seedling growth, *B. amyloliquefaciens* strain DB2 suspensions were found to enhance the growth of plants compared with the control. The preliminary experiments were performed with wheat inoculated with DB2 suspensions planted in the greenhouse. These wheat seedings were more developed than noinoculated ones, particularly in the root system. It has proved that our strain can increased plant and root length, plant fresh and dry weight. Furthermore, the culture filtrate also alleviated the unfavorable environmental conditions in LB medium. We tentatively speculate that this may be associated with the containing plant growth promoting substances in culture filtrate.

**Figure 9:**
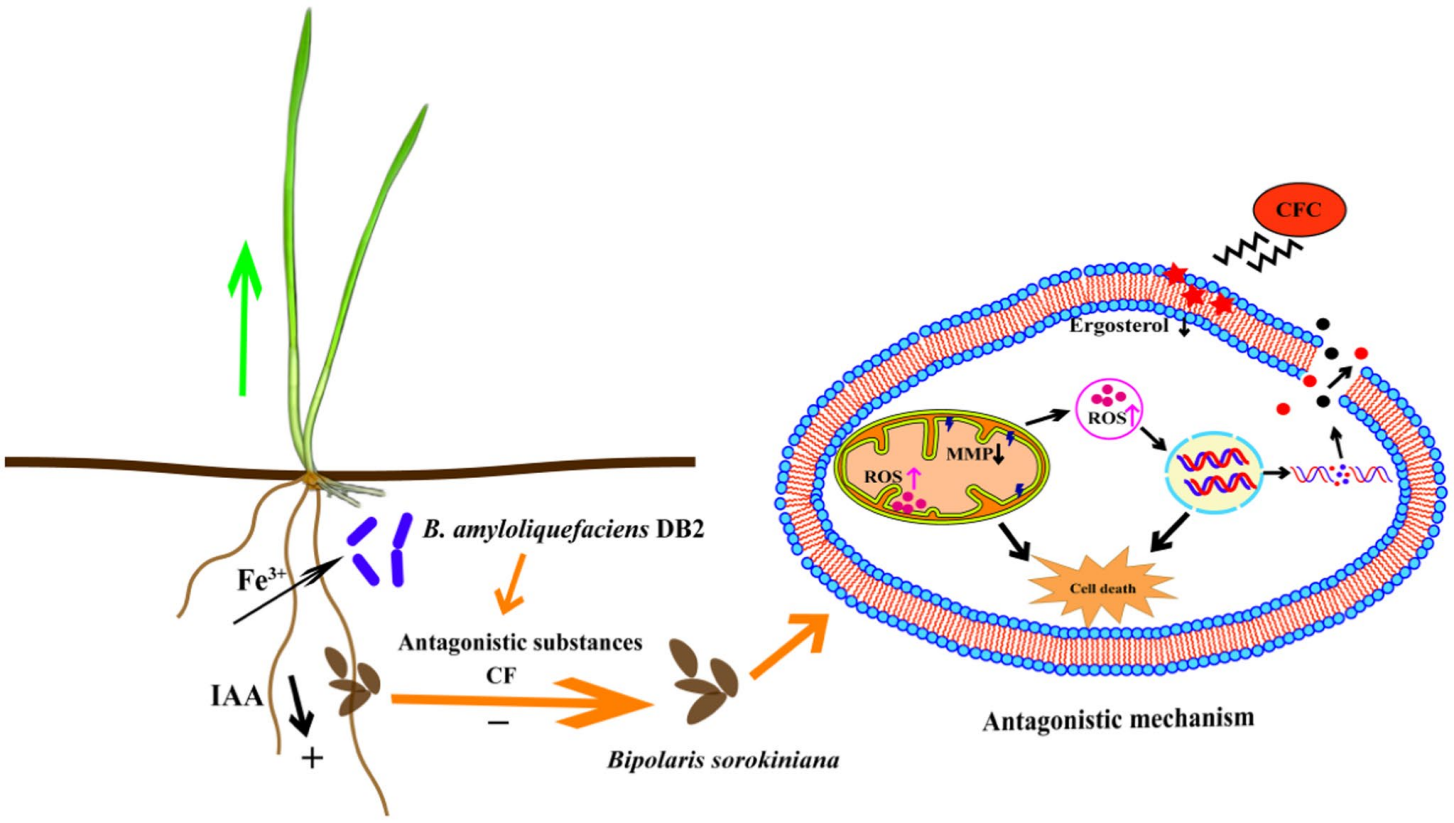
Proposed model showing that *Bacillus amyloliquefaciens* DB2 can promote wheat seedlings growth and underlying mechanisms of antagonistic strain DB2 against *Bipolaris sorokiniana*.

Overall, antagonistic strain *B. amyloliquefaciens* DB2 could be taken as the potential BCAs for *B. sorokiniana* control. It is necessary to reveal the mechanism of biocontrol bacteria on plant diseases for developing effective BCAs. The underlying action mechanisms are concluded including apoptosis by disrupting the cell membrane integrity, decreasing the MMP and accumulation of ROS in the mycelia cell, thereby inhibiting the growth of pathogen, and producing of growth-promoting substances for wheat growth and health (Figure 9). For better defining its direction of action, purification and identification of the antifungal and growth-promoting substances are necessary to determine to further study the specific effects on the pathogen.

## 5. Conclusion

In the current study, we investigated a biocontrol strain that could be used as an alternative agent for controlling *B. sorokiniana*, and the experiment results also enhanced our comprehensive understanding toward the possible antifungal mechanisms of the *CF* of *B. amyloliquefaciens* DB2*. In vitro* antagonistic assay showed that culture filtrate of the bioactive bacterium could disrupt the cell membrane integrity, decrease the MMP and accumulate ROS in the mycelia cell to inhibit the growth of fungal pathogen. The strain DB2 could produce extracellular enzymes, protease, amylase, siderophore and IAA. Furthermore, it is evident that the culture filtrate successfully suppressed or reduced disease symptoms and the strain DB2 suspension had a significant promotion of wheat seedlings growth. So, it may be concluded that the strain *B. amyloliquefaciens* DB2 could be utilized for biocontrol management (*B. sorokiniana*) program in the sustainable development of the agriculture.

## Data availability statement

The datasets presented in this study can be found in online repositories. The names of the repository/repositories and accession number(s) can be found at: https://www.ncbi.nlm.nih.gov/, MZ664342.1.

## Author contributions

PL: conceptualization, methodology, data curation, formal analysis, and writing original draft. YY: conceptualization, funding acquisition, project administration, resources, supervision, and writing and revising. YH, LZ, XR, and SJ: methodology, investigation, and validation. LC, ZH, and YL: data curation and formal analysis. All authors contributed to the article and approved the submitted version.

## Funding

This work was supported by the Henan Provincial Science and Technology Major Project (221100110100), China Agriculture Research System of MOF and MARA (CARS-03), and the Innovative Funds Plan of Henan University of Technology (2020ZKCJ23).

## Conflict of interest

The authors declare that the research was conducted in the absence of any commercial or financial relationships that could be construed as a potential conflict of interest.

## Publisher’s note

All claims expressed in this article are solely those of the authors and do not necessarily represent those of their affiliated organizations, or those of the publisher, the editors and the reviewers. Any product that may be evaluated in this article, or claim that may be made by its manufacturer, is not guaranteed or endorsed by the publisher.

## Footnotes

^1^ http://www.Ncbi.nlm.nih.gov
